# Antipodean Perspectives—Aged Care Nursing and the Multifaceted Role of the Aged Care Nurse

**DOI:** 10.3390/nursrep12030062

**Published:** 2022-08-30

**Authors:** Rajkumar Cheluvappa, Selwyn Selvendran

**Affiliations:** 1 Nursing and Midwifery, Australian Catholic University, Watson, ACT 2602, Australia; 2 Department of Surgery, St George Hospital, Kogarah, NSW 2217, Australia

**Keywords:** act, activities of daily living, aged care, aged care assessment team, aged care quality and safety commission, ageing, aging, Australia, Australian, dementia, elder abuse, healthy ageing, healthy aging, law, legislation, nurse, nursing, nursing and midwifery board of Australia, registered nurse

## Abstract

Healthy ageing refers to the development and maintenance of the functional ability of ageing individuals. Aged care nurses provide nursing care to elderly individuals and usually work in aged care residential facilities, nursing homes, home care services, and/or hospital departments. The registered nurse working in the aged care sector has several important roles. Key roles cover both therapeutic and preventative paradigms, as discussed in this paper. The aged care nurse is also “tasked with” holistic patient-centred care and the promotion of healthy ageing via advocacy and sociocultural roles. This paper examined, described, and analysed the multifaceted role of an aged care nurse from an Australian perspective. We conducted meticulous searches using PubMed, Google Scholar, government guidelines, authoritative body regulations, quality control guidelines, and government portals pertaining to aged care nursing in Australia. This paper relied upon the information garnered from publications, reports, and guidelines resulting from these searches and analyses. Multiple aspects of healthy ageing and holistic aged care nursing are discussed. The key roles of the aged care nurse are enumerated next, in accordance with the code of conduct from the Nursing and Midwifery Board of Australia (NMBA). The NMBA promotes evidence-based, culturally sensitive, consultative, holistic aged care clinical practice that includes input from care recipients, their decision makers, and/or their health care providers. The difficult issue of loneliness is discussed with strategies to ameliorate aspects of this. Good social networks, community interactions, meaningful friendships, and participation in personalised spiritual/religious practices improve the quality of aged care. The key topic of elder abuse and its forms are discussed apropos of aged care nursing. Healthy ageing is promoted by identifying and reporting elder abuse at the earliest. Current Australian law and recent federal legislation changes pertaining to aged care nursing are discussed next. As a result of these legislation changes, several new quality control imperatives (for aged care organisations/facilities) under the Aged Care Quality and Safety Commission (ACQSC) have been implemented. Residential and flexible aged care providers should now have robust ongoing documentation and a well-developed behaviour support plan (BSP) for each care recipient who currently requires or may require restrictive practices, which must be reported under the new serious incident reporting scheme (SIRS). Various strategies to promote healthy ageing and approaches to communicate effectively with aged care recipients are also discussed. Healthy ageing is promoted when age care recipients are empowered with making their own autonomous choices in “major and minor” aspects of life. Finally, approaches to optimise quality aged care nursing care are discussed. The Roper–Logan–Tierney model is one of the models used to assess and optimise nursing care. This is premised on the capability of an ageing individual to accomplish 12 basic activities of daily living.

## 1. Healthy Ageing and Holistic Aged Care Nursing

The World Health Organization connects the concept of healthy ageing to the functional ability of an ageing individual, and its careful development, nurturing, maximisation, and maintenance [1]. Functional ability is referred to as “having the capabilities that enable all people to be and do what they have reason to value” [1]. In other words, functional ability covers the meeting of basic activities of daily living, starting/maintaining relationships, and multi-faceted contributions to the community [1]. It also includes the close interactions between the inner abilities of an ageing individual and the society in which they live, inclusive of family members, pet animals, housing, neighbours, friends, marketplaces, etc. [1].

Holistic health in ageing includes an ageing individual’s sensory components, psychological elements, diseases (especially chronic conditions), biological predispositions, etc. An aged care nurse ought to develop and nurture holistic, but communicative and consultative personalised care approaches [2,3]. Cognition improves and health declines slowly when ageing individuals participate frequently in social groups, group events, and community programs [4]. Individualised spiritual/religious activities and participation in preferred rituals improve cognition and the quality of life in ageing individuals [5,6]. Art and music improve an ageing individual’s mood, affect, and overall functional ability [7]. Consultative care planning and approaches may frequently involve the ageing patient’s relatives, well-wishers, clinicians, and friends [2,3].

The aged care nurse is expected to reflect and practice towards improving and providing consistent, quality care for ageing individuals/clients/patients with maladies [2,3]. As permitted by the law, rules, and patient care plans, an aged care nurse is expected to offer choices to ageing patients or aged care residents whilst assisting in their day-to-day activities, meals, modes of transportation, physical mobilisation, community outings, etc. [3,8]. The conferring of choices such as these would in turn empower the ageing clients/patients. When ageing individuals are empowered thus, there may be marked improvements in various facets of their functional ability [9]. Improvements in functional ability, in turn, promote healthy ageing.

Whilst it is theoretically possible to offer holistic patient-centred aged care nursing with substantial involvement, it is necessary for the aged care nurse to chart out and maintain professional boundaries, without compromising the empathy, integrity, and quality of care [2,10]. This would indeed be an integral part of the “art of aged care nursing”.

An aged care nurse is expected to maintain a keen eye on an ageing patient’s clinical conditions including exacerbations of pre-existing/chronic health issues, cognitive deterioration, overall health decline, and new clinical manifestations. Developing clinical acumen as when to intervene, not react, or escalate in a confronting clinical or behavioural patient scenario is an important aspect of aged care nursing [2,11].

Risk minimising, hazard management, community adjustments, and commensurate environmental modifications are within the ambit of the role of an aged care nurse [2,11]. This is ideally conducted by factoring in the current and projected cognitive, functional, and sensorimotor decline in ageing patients, and executing appropriate social adjustments and environmental modifications towards risk minimisation and hazard management [2,11].

## 2. Research Approach

Information was gathered from publications, reports, and guidelines resulting from PubMed, Google Scholar, quality control guidelines, and government portals pertaining to aged care nursing in Australia. The key search words we used included single or multiple combinations of act, activities of daily living, aged care, aged care assessment team, aged care quality and safety commission, ageing, aging, Australia, dementia; elder abuse, healthy ageing; healthy aging, law, legislation, nurse, nursing, nursing and midwifery board of Australia, and registered nurse. Publications that defined and promoted healthy ageing and quality holistic aged care nursing were perused for analysis with additional consultation from the Code of Conduct (COC) from the Nursing and Midwifery Board of Australia (NMBA). Papers pertaining to topics of concern in ageing were perused and referenced, especially with reference to recent relevant Australian legislation changes. Our meticulous searches resulted in about 30 publications and around 10 government/official reports. Being a brief report and analytical treatise, this paper does not fall under the purview of the Preferred Reporting Items for Systematic Reviews and Meta-Analyses (PRISMA) as it is not a systematic review or a meta-analysis.

## 3. The Key Roles of the (Aged Care) Nurse Outlined by the Nursing and Midwifery Board of Australia

The Code of Conduct (COC) from the Nursing and Midwifery Board of Australia (NMBA) enumerates and summarises the foundational roles of the registered nurse in establishing, nurturing, and maintaining clinical practices ensuring healthy ageing [12].

The NMBA COC promotes evidence-based holistic clinical practice, which is also consultative with patients and their well-wishers (NMBA COC 2) [12]. The NMBA COC underscores autonomous culturally sensitive patient decision-making, with the conferring of dignity, respect, and confidentiality (NMBA COC 3) [10,12]. Moreover, the NMBA COC emphasises the equitable and appropriate involvement of family, friends, and community well-wishers in nursing practice (NMBA COC 7) [12]. Towards promoting quality holistic aged care, aged care nurses should develop a firm grasp of available health services including primary care facilities, the closest emergency services, speciality referral facilities, secondary care centres, tertiary care hospitals, general practitioners who visit nursing homes, and local community health services [2,3,8,11]. Commensurate with items in the NMBA COC, an aged care nurse should not compromise facts, alter details, or falsify records at any level, but should develop empathetic, good quality record keeping [2,3,8,11].

## 4. Loneliness in Ageing and Elder Abuse

In the natural process of ageing, isolation and loneliness is common, and may be more noticeable in a large proportion of ageing individuals [13]. It has been repeatedly shown that good social networks and community interactions diminish the morbidity of maladies and mortality in ageing individuals [4,14]. Social interactions, community programmes, and planned outings empower the ageing individual, provide the interdependent feeling of being needed, and decrease the magnitude of cognitive declines in ageing individuals when compared to their counterparts who experience more isolation [6,15].

It is noteworthy that differences in the nationalities, ethnicity, personalities, and cultures can significantly impact the health outcomes and behaviours of ageing individuals [16,17]. Due to this, the mental well-being and health in ageing individuals are enhanced when they are empowered with making their own autonomous choices in picking their social activities, friends circles, menu, clinical care, personal care, home modifications, travel options, etc. [3,8,10]. It has been shown that spiritual activities, art, music, and traditional practices improved the health indicators in ageing Americans by enhancing their zest for life [7]. Similarly, it has been demonstrated that self-identifying spiritual practices improve the health of ageing Indigenous Australians [18].

Although the term “elder abuse” is more often used today, there is no precise definition. Elder abuse is held to refer to the maltreatment of individuals above 60 years of age. The type of abuse varies widely and includes psychological abuse, sexual abuse, financial exploitation, physical abuse, and direct violence [19].

Financial abuse, plain cheating, mental abuse, and violence can occur across different types of facilities including hospitals, respite centres, rehabilitation units, social outreach programmes, aged care facilities, and even in the ageing individual’s home [20]. Unfortunately, elder abuse is frequently inflicted by a close relative [20]. Community settings are places where elder abuse frequently occurs [21].

Elder abuse is often predictable and commonplace. Therefore, healthy ageing can be promoted by identifying and reporting elder abuse at the earliest; and maximally preventing them when possible by putting in appropriate measures [21]. Aged care nurses should watch for both subtle and conspicuous indications of elder abuse including behaviour changes, and escalate it immediately to initiate punitive measures against the perpetrator(s) and minimise future occurrences of similar abuse [21].

Except in the Australian Capital Territory (ACT), no specific elder abuse laws exist across Australia. The ACT Elder Abuse legislation was passed in 13 August 2020, and came into force in April 2021 [22]. The identification and reporting of elder abuse would be an essential component of an aged care nurse’s duties as it empowers the aged care recipients [21].

## 5. Australian Law and Recent Federal Legislation Changes Pertaining to Aged Care Nursing

While civil liability and discrimination laws apply to aged care nursing, the Aged Care Act 1997 (federal law) is the foundational legislation covering aged care services funded by the government [23]. This law regulates government-funded aged care and covers the funding, provider approvals, compliance, resident rights, and aged care quality control. A set of 17 legislative principles were published in 2014 to explain the Aged Care Act 1997 further [24].

Other Australian laws covering related aspects of aged care include the Aged Care (Living Longer Living Better) Act 2013, Aged Care (Transitional Provisions) Act 1997, Aged Care (Accommodation Payment Security) Levy Act 2006, Aged Care (Accommodation Payment Security) Act 2006, Aged Care Legislation Amendment (Increasing Consumer Choice) Act 2016, and the Aged Care Amendment (Red Tape Reduction in Places Management) Act 2016 [24].

The Aged Care Quality and Safety Commission (ACQSC) is a federal non-corporate body that protects, promotes, and nurtures the well-being, safety, holistic health, and living standards of individuals using aged care sector services [10,25]. The ACQSC was established by the Aged Care Quality and Safety Commission Act 2018 (Act) [10,24]. This act/law establishes the ACQSC and empowers the responsible federal minister to craft rules covering or/and effecting the processes of the ACQSC [17]. In turn, the processes and functions of the ACQSC are overseen and mediated by the Aged Care Quality and Safety Commission Rules 2018 (Rules), which goes hand in hand with the Aged Care Quality and Safety Commission Act 2018 (Act) [20].

In 2021, the *Aged Care Act 1997* was amended to action three measures stemming from recommendations of the ACQSC as well as the “*Independent Review of Legislation Provisions Governing the use of Restraint in Residential Aged Care*” [26]. The ACQSC strove to minimise or negate the misuse of restrictive practices towards lessening the risks to the care recipients’ health and safety [26,27]. A new definition of restrictive practices was introduced in the Act to bring care standards into line with the disability care [27]. Commensurate changes were also made to the *Quality of Care Principles 2014* to explain these new changes incorporated in the Act.

Residential and flexible aged care providers should now have robust ongoing documentation and a well-developed behaviour support plan (BSP) for each resident in their facilities who currently requires or may require restrictive practices as an integral part of their care plan [28]. The BSP is to be integrated in the care plan. The BSP does not replace the care plan. The ACQSC describes the ideal BSP as [27]:

*“a plan for a consumer, made in accordance with the [Quality of Care] Principles, that sets out information regarding any changed behaviours (also known as ‘behaviours of concern’) for that consumer, including all alternative strategies which have been tried prior to the use of any restrictive practice for that consumer. The Principles set out the responsibilities of the provider relating to Behaviour Support Plans, including assessments and documentation which should be prepared and included.”*

The ACQSC requires that aged care providers institute a relevant/proper clinical governance framework [29]. The ACQSC also requires their consultation with care recipients (and/or their decision maker) and their health care providers before preparing, reviewing, or revising their BSPs [28]. If any restrictive practice is utilised, the care recipient must be consistently monitored for distress, adverse effects, harm, mood/behaviour variations, side effects, and deterioration in the magnitude/quality of daily activities [26]. The ACQSC monitors how compliant aged care providers are to these legislative changes and is endowed with the authority to enforce regulatory action (e.g., Restrictive Practices Compliance Notices) if lapses are detected and determined [26]. The ACQSC indicates that the utilisation of a BSP-unauthorised restrictive practice on an ageing client under aged care services is a reportable incident under the *Serious Incident Reporting Scheme* (SIRS) [30].

## 6. Australian Government Funding for Aged Care

Ageing individuals requiring increasing levels of care usually contact “My Aged Care”, who arrange a free assessment with the local Aged Care Assessment Team (ACAT) to determine their suitability for placement in residential aged care facilities. Although waiting lists may be long, ageing individuals can apply for subsidised home care packages to access basic day-to-day work at home [31]. Higher mortality rates, in proportion to the length of wait lists, have been noted in ageing clients waiting to enter residential aged care facilities [32]. Health ageing also requires quicker clearing of these backlogs. Stating the obvious, the faster and simpler home care or residential care services are accessed, the better for ageing individuals (and their functional abilities) looking to utilise these services [33].

## 7. Promotion of Healthy Ageing and Optimisation of Quality Aged Care Nursing

Aspects of physical, social, and psychological well-being must all be factored in towards providing individualised care and promoting healthy ageing [2,3]. Emphasis should be placed on well-planned community interactions, the provision of quality interpersonal interactions, the need for meaningful friendships, and the inclusion of personalised spiritual/religious practices [5,6]. When these physical, social, and psychological paradigms are integrated in planning and executing aged care services, healthy ageing can be successfully promoted [34]. Several structured ageing health care programmes and resources promoting holistic patient-centred well-being are available [2,8].

It is well-known that there are long waiting times and backlogs in the allocation of government funding for residential aged care placement and home care packages [31,32]. It is also on record that the mortality, morbidity, and maladies increase during this waiting period [31,32]. To minimise the exacerbation of existing health issues and death during waiting, strengths-based nursing may be utilised to mobilise and informally roster the well-wishers, family, and friends of ageing individuals to assist in their basic needs and promote their healthy ageing [35,36].

Ageing individuals often suffer impaired cognition, cardiovascular issues, neurological deficits, chronic pain, osteoporosis, hearing loss, etc., leading to communication lapses. Moreover, sociological differences, ethnic origin, linguistic differences, religious imperatives, and personal convictions are likely to widen the communication divide. Therefore, aged care nurses should develop flexibility in speaking, good communication abilities, and the versatility to quickly take note of an aged care patient’s behavioural and verbal changes and adapt their communication approaches accordingly [2,3,11]. The use of authorised, trained interpreters when necessary are likely to be useful in certain scenarios. Introducing and familiarising ageing individuals to the use of simple communication devices, electronic gadgets, and medical devices (e.g., glucometers) are likely to enhance the accuracy, timeliness, appropriateness, and precision of communication in ageing individuals [2]. This is likely to promote healthy ageing by imparting the spirit of “being included”, and the satisfaction of having used electronic gadgetry to assess oneself and to communicate or reach out to others [2].

The quality of aged care nursing is improved by empowering the aged care recipient to participate in the choice of care items [6,10]. Quality aged care nursing is buttressed by the meticulous planning and provision of patient-centred holistic health care [2]. Constant personal/electronic monitoring, observations, and appropriate reporting/escalation of declining health or the onset of new clinical presentations improve the quality of aged care nursing [11].

In 2020, the ACQSC promulgated eight quality standards to qualitatively measure (against a denominator of four bars) how aged care homes perform, and to ascertain whether they have and maintain good quality aged care [37]. The first one is the maintenance of consumer dignity and informed life and health choices [10,37] The second standard pertains to the inclusion of the aged care recipient in continuous assessment and planning towards obtaining the care services the recipient wants [37]. The third and fourth standards target safe, preferred, and appropriate care, services, and supports required for daily living [37]. The fifth to eighth standards pertain to the organisation and functional structure including the service environment, action on complaints/feedback, efficient human resources, and quality organisational governance [37].

There are several evidence-based models used worldwide to qualitatively assess and optimise nursing care towards improving the quality of aged care nursing [38]. One such evidence-based model, which is widely used is the Roper–Logan–Tierney model. The Roper–Logan–Tierney model is premised on the capability of an ageing individual to accomplish 12 basic activities of daily living covering communication abilities, environment safety, oral intake and elimination, work and sleep, personal care, thermoregulation, physical activity, sexuality, and dying [39].

## 8. Conclusions

This paper covers information pertaining to the practice of quality aged care nursing. Supported with evidence, we initially discussed healthy ageing in the context of functional ability and holistic, consultative, patient-centred, holistic nursing care. The crucial role of the registered aged care nurse was elaborately discussed under various topics. The aged care nurse not only has a pathology-oriented therapeutic and preventative role, but also a supportive, empowering, edifying niche role in home-based and residential aged care. The key roles of the aged care nurse according to the Code of Conduct from the Nursing and Midwifery Board of Australia were touched upon. This paper pinpoints pertinent items of concern and important areas of focus in the aged care milieu, which are also important topics for the teaching and research therein. The painful issues of loneliness in ageing and elder abuse were discussed with evidence-based strategies to minimise these and the negative impact of these. Current Australian law and recent changes/mandates apropos of aged care nursing were discussed next. The promotion of healthy ageing and approaches to communicate effectively with aged care recipients were deliberated on. Finally, approaches to optimise, maintain, and improve quality aged care nursing care were discussed including the Roper–Logan–Tierney model and the ACQSC quality standards.

## Abbreviations

ACAT—Aged Care Assessment Team; ACQSC—Aged Care Quality and Safety Commission; COC—Code of Conduct; NMBA—Nursing and Midwifery Board of Australia.
